# How to balance the bioinformatics data: pseudo-negative sampling

**DOI:** 10.1186/s12859-019-3269-4

**Published:** 2019-12-24

**Authors:** Yongqing Zhang, Shaojie Qiao, Rongzhao Lu, Nan Han, Dingxiang Liu, Jiliu Zhou

**Affiliations:** 1 School of Computer Science, Chengdu University of Information Technology, Chengdu, 610225 China; 2 School of Computer Science and Engineering, University of Electronic Science and Technology of China, Chengdu, 610054 China; 3 School of Software Engineering, Chengdu University of Information Technology, Chengdu, 610225 China; 4 Software Automatic Generation and Intelligent Service Key Laboratory of Sichuan Province, Chengdu University of Information Technology, Chengdu, 610225 China; 5 School of Management, Chengdu University of Information Technology, Chengdu, 610103 China; 6 School of Cybersecurity, Chengdu University of Information Technology, Chengdu, 610225 China

**Keywords:** Imbalanced data, Pseudo-negative sampling, Pearson correlation coefficients, Max-relevance, Min-redundancy

## Abstract

**Background:**

Imbalanced datasets are commonly encountered in bioinformatics classification problems, that is, the number of negative samples is much larger than that of positive samples. Particularly, the data imbalance phenomena will make us underestimate the performance of the minority class of positive samples. Therefore, how to balance the bioinformatic data becomes a very challenging and difficult problem.

**Results:**

In this study, we propose a new data sampling approach, called pseudo-negative sampling, which can be effectively applied to handle the case that: negative samples greatly dominate positive samples. Specifically, we design a supervised learning method based on a max-relevance min-redundancy criterion beyond Pearson correlation coefficient (MMPCC), which is used to choose pseudo-negative samples from the negative samples and view them as positive samples. In addition, MMPCC uses an incremental searching technique to select optimal pseudo-negative samples to reduce the computation cost. Consequently, the discovered pseudo-negative samples have strong relevance to positive samples and less redundancy to negative ones.

**Conclusions:**

To validate the performance of our method, we conduct experiments base on four UCI datasets and three real bioinformatics datasets. According to the experimental results, we clearly observe the performance of MMPCC is better than other sampling methods in terms of Sensitivity, Specificity, Accuracy and the Mathew’s Correlation Coefficient. This reveals that the pseudo-negative samples are particularly helpful to solve the imbalance dataset problem. Moreover, the gain of Sensitivity from the minority samples with pseudo-negative samples grows with the improvement of prediction accuracy on all dataset.

## Background

The work is motivated by the real-world requirement in bioinformatic data processing: it is very common that negative samples greatly dominate positive samples, and this phenomena is called data imbalance problem. In general, we cannot achieve genetic data mining with limited positive samples. So, we think that: whether we could use positive samples by mixing pseudo-negative data (which is classified to be negative data, but they are similar to positive samples with the maximum relevance and they have the minimum redundancy with negative samples) to predict the categories of samples. Because of the lack of enough positive samples, the biologist cannot perform experiments. Consequently, some positive samples cannot be identified or categorised as negative samples which can be viewed defined as pseudo-negative samples. So how to select these pseudo-negative samples will be an alternative method to solve the imbalanced data problem in bioinformatics.

In the post-genome era, with the wide application of various high-throughput technologies, biological data has rapidly increased [1, 2]. Machine learning technology can be applied to discovery important information for understand complex biological processes from large-scale biological data [3–9]. However, imbalanced data is a very common phenomenon in the real dataset (where the positive sample is the minority class). Many bioinformatics applications require class imbalance learning, such as gene expression data [10, 11], protein-DNA binding data [12, 13], *N*^6^-methylation sites in mRNAs [14], splice sites prediction [15], prediction of microRNAs [16], prediction of protein interaction [17–21], transcription factor binding sites prediction [22, 23] and so on. In this scenario, the performance of the minority classes can be greatly underestimated [24].

To the best of our knowledge, researchers have proposed some strategies to degrade the influence of imbalance data. These existing methods can be classified into data-level approaches and algorithmic-level approaches [25, 26]. In regard of data-level approaches, re-sampling techniques are employed to balance the sample space w.r.t. an imbalanced dataset in order to alleviate the negative effect of the skewed distribution of samples in the learning process. Resampling methods are very commonly-used approach because they are independent of classifiers. Resampling techniques can be classified into three categories depending on the method used to balance the proportion of positive and negative samples: (1) over-sampling: eliminating the negative effect of skewed distribution by generating new samples of minority class. Two widely-used approaches to generate minority samples are Random Over-Sampling (ROS) which randomly duplicate the minority samples, and SMOTE. (2) Under-sampling: balance the data by discard the samples from the majority class. The simplest yet most effective method is Random Under-Sampling (RUS) which involved the random elimination of majority class examples [27]. RUS deals with the class imbalance problems in an effectively fashion. (3) Hybrid methods: these are a combination of the over-sampling and under-sampling method. The commonly-used algorithmic-level approach is cost-sensitive learning method which assigns higher costs to the minority class [28, 29].

However, RUS often loses some important classification information and ROS is time-consuming and often results in the phenomenon of overfitting. So, it is essential to propose advanced data sampling approaches to maintain the structure of groups and generate new data according to its underlying distribution.

To overcome the problems caused by the imbalanced bioinformatic data, we first propose the pseudo-negative sampling approach based on Max-relevance and Min-redundancy Pearson correlation coefficient (called MMPCC). In the MMPCC approach, Pearson correlation coefficients are used to measure the similarity between positive and negative samples and the coefficients are learned from positive and negative samples based on the max-relevance and min-redundancy criteria. The new algorithm can discover the pseudo-negative samples which may be viewed as positive samples, but their labels are negative. This proposed sampling approach aims at alleviating the imbalanced ratio. The experiments are applied on two UCI data and three real-life bioinformatics data.

**Contribution:** The original contributions of this study can be summarized as follows.

1) We propose a concept of pseudo-negative samples and present a pseudo-negative sampling method which is based on the max-relevance and min-redundancy Pearson correlation coefficient in supervised learning. In particular, both positive and negative samples are taken into full consideration in order to find optimal pseudo-negative samples.

2) We use an incremental searching method for calculating the coefficient of positive and negative samples, which can avoid the high computational cost in selecting the subsets of pseudo-negative samples.

3) We conduct extensive experiments and the results demonstrate the advantage of the MMPCC method for handling the imbalanced bioinformatic data.

## Methods

### Pseudo-negative sampling method

Although pseudo-negative samples are viewed to be negative, but they are similar to positive samples with the maximum relevance and they have the minimum redundancy with negative samples. The key idea of pseudo-negative sampling approach is to select a subset from the negative samples and classify them into positive class by the method of max-relevance and min-redundancy on Pearson correlation coefficient in the phase of training. The formal definition of pseudo-negative samples is given as follows.

#### Definition 1 (Pseudo-negative samples).

Given a positive data set *S*^+^=$\{(x^{+}_{1},y^{+}_{1}),(x^{+}_{2},y^{+}_{2}),...,(x^{+}_{m},y^{+}_{m})\}$, a negative data set *S*^−^=$\{(x^{-}_{1},y^{-}_{1}),(x^{-}_{2},y^{-}_{2}),...,(x^{-}_{n},y^{-}_{n})\}$, then a pseudo-negative data set is represented by *S*^∗^=$\{(x^{*}_{1},y^{*}_{1}),(x^{*}_{2},y^{*}_{2}),...,(x^{*}_{l},y^{*}_{l})\}$, where *m* is the total number of positive data, *n* is the total number of negative data, *m*≪*n*, and *l* is the number of pseudo-negative samples.

The purpose of our method is to identify the pseudo-negative sample set *S*^∗^ (which might contain *l* samples) based on *S*^+^ and *S*^−^, where *l*<*m*.

One of the famous sequential search methods is the incremental sample search algorithm, and we employ it in the study. To achieve the incremental sample searching, the pseudo-negative sample set starts from $S^{*}_{0}=\varnothing $, and a quantitative criterion $Q(S^{*}_{i})$ is used to measure the similarity of samples in $S^{*}_{i}$.

In each round of searching, a sample *S*^∗′^ would be added in the sample set $S^{*}_{k}$.

1 $$ S_{k}^{*}=S_{k-1}^{*}\cup S^{*\prime}  $$

where

2 $$ S^{*\prime}= \underset{S_{k-1}^{*}\cap S^{*\prime}=\varnothing}{argmax \ Q(S_{k}^{*})}\  $$

$Q(S_{i}^{*})$ plays an important role in the sample selection, which can be defined with different requirements. The validation accuracy is utilized to evaluate the new sample subsets. In this study, the metric of Eq. 3 is utilized to evaluate the similarity of samples in $S_{k-1}^{*}$ and *S*^∗′^, and the corresponding quantitative criterion is given by the following equation:

3 $$  Q(S_{i}^{*})=A\left(S_{k-1}^{*}\cup S^{*\prime}\right)  $$

where *S*^∗′^ is a potential pseudo-negative sample and $S^{*}_{k-1}$ is the pseudo-negative sample set, and *A* represents the validation accuracy.

In this study, we employ the Pearson correlation coefficient between samples in order to select a new sample. $Q(S_{i}^{*})$ can be transformed to be the following equation:

4 $$ Q(S_{i}^{*})=P\left(S_{k-1}^{*}\cup S^{*\prime}\right)  $$

The details of calculating the Pearson correlation coefficient are given in the following.

### Max-relevance and min-redundancy on pearson correlation coefficient

Pearson correlation coefficient (PCC) [30] is defined on the covariance matrix, which is a method to evaluate the strength of the relationship between two vectors. In general, the coefficient between two vectors *α*_*i*_ and *α*_*j*_ is defined as follows:

5 $$ P(\alpha_{i},\alpha_{j})=\frac{cov(\alpha_{i},\alpha_{j})}{\sqrt{var(\alpha_{i})\times var(\alpha_{j})}\qquad}\qquad  $$

According to the max-relevance, PCC beyond negative sample and positive sample are formalized as follows:

6 $$ D(S^{-}_{i},S^{+}_{j})=P(S^{-}_{i},S^{+}_{j})  $$

where $S^{-}_{i} \in S^{-}, i \in N, S^{+}_{j} \in S^{+}$ and *j*∈*M* agreeing with the max-relevance criterion. The most relevant feature set can be obtained by maximizing $D(S^{-}_{i},S^{+}_{j})$.

7 $$ S_{max} = argmax \ D(S^{-}_{i},S^{+}_{j})  $$

Based on the min-redundancy criterion, the samples could be selected by the following equation:

8 $$ R = \frac{1}{|S^{*}|^{2}}\sum P(S^{-}_{i},S^{*}_{k})  $$

where $S^{-}_{i} \in S^{-}$ and $S^{*}_{k}\in S^{*}$,

9 $$ S_{min} = argmin \ \{R\}  $$

In terms of incremental search method, an operator *Ψ*(*D*,*R*) is defined in Equation 10 in order to optimize the max-relevance and min-redundancy information. The best selected sample *S*^∗′^ is given as follows:

10 $$ \Psi=D-R  $$

11 $$ S^{*\prime}=argmax \ \Psi(D,R)  $$

Assume we have the sample subsets $S^{*}_{k-1}$ which have *k*-1 samples. In the next step of searching, the *k*^*th*^ sample is obtained from the sample subsets $\{S^{-}-S^{*}_{k-1}\}$. Then, $S^{*}_{k}$ can be calculated by Eq. 12 based on *Ψ*(*D*,*R*).

12 $$ S^{*}_{k}={argmax}\left[P(S^{-}_{i},S^{+}_{j})-\frac{1}{k-1}\sum_{S^{*}_{k} \in S^{*}_{k-1}} P(S^{-}_{i},S^{*}_{k})\right]  $$

where $S^{-}_{i}\in \{S^{-}-S^{*}_{k-1}\}$ and $S^{+}_{j} \in S^{+}$.

### The proposed pseudo-negative sampling algorithm

**Fig. 1:**
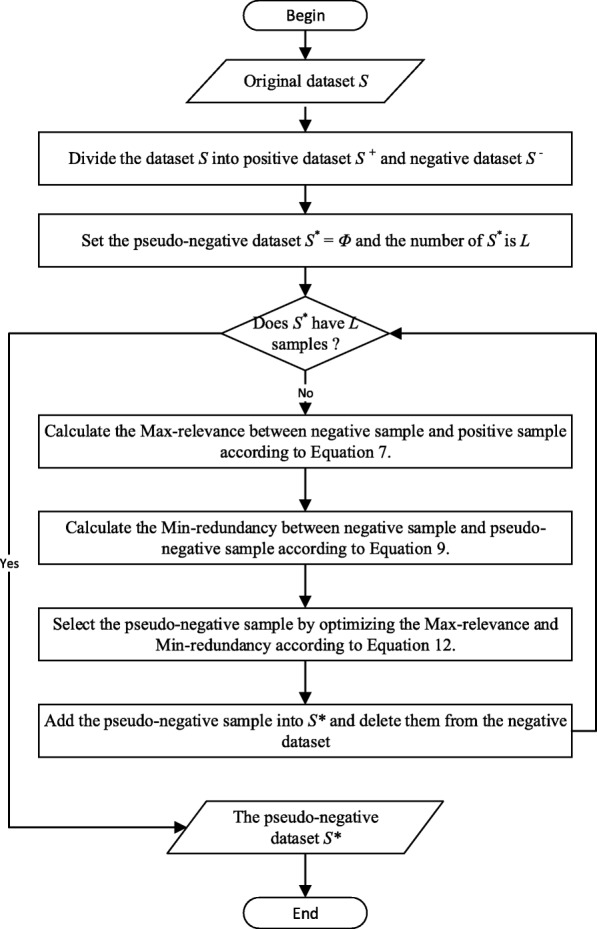
A flow chart of MMPCC algorithm


Based on the aforementioned preliminaries, we propose a pseudo-negative sampling algorithm based on the max-relevance and min-redundancy on Pearson correlation coefficient, which is called MMPCC. The detail of the MMPCC algorithm is presented in Algorithm 1 and the flow chart is shown in Fig. 1.

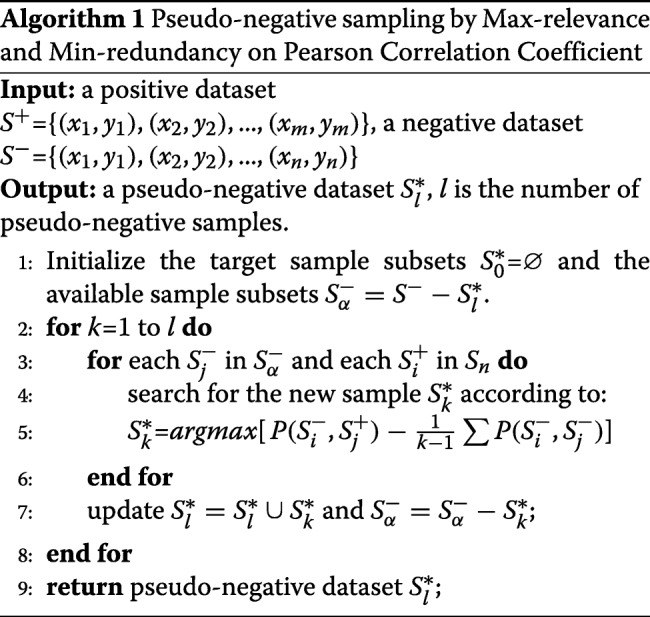


As described in Algorithm 1, the selected pseudo-negative samples can be updated step by step. Firstly, the max-relevance between the negative sample and the positive sample is calculated by Equation 7 in order to choose candidate pseudo-negative samples. Then, the new selected sample will be identified based on the min-redundancy of samples in the selected pseudo-negative subsets by Equation 9. Lastly, the new sample will be identified to be pseudo-negative sample by Equation 12.

It is worthwhile to note that *l* is specified by experts in order to determine how many pseudo-negative samples should be inserted into the positive sample set.

The computational complexity of MMPCC, MAXR and MINR includes two parts: the computation of similarity matrices and the computation of sample ranking. The operator *ψ*_*MAXR*_ can be obtained via Equation 7, the operator *ψ*_*MINR*_ can be calculated by Equation 9 and the MMPCC model be figured out by Equation 12.

As for MAXR, the computation of Pearson correlation coefficient of all pairwise negative data and positive data requires the complexity of *O*(*n*∗*m*∗*f*), where *n* is the number of negative data, *m* is the number of positive data and *f* is the number of attributes of each data. As for MINR, the computation complexity is *O*(*n*∗*l*∗*f*), where *l* is the number of pseudo-negative samples. Therefore, the computation complexity of MMPCC is the sum of MAXR and MINR, that is, *O*(*n*∗*m*∗*f*+*n*∗*l*∗*f*).

### Classification methods

#### Random forests

The classifier of Random forests [31, 32] is an ensemble learning method, which works by constructing a multitude of decision trees at training time and outputting the class that is the mode of the classes (classification) or mean prediction (regression) of the individual trees. Random decision forests correct for decision trees’ habit of overfitting to their training set.

#### Neural networks

A neural network [33] is composed of several simple "neurons", and the output of a neuron will be the input of another. The connections of the biological neuron are modeled as weights. A positive weight reflects an excitatory connection, while negative values mean inhibitory connections. All inputs are modified by a weight and summed. This activity is referred as a linear combination. Finally, an activation function controls the amplitude of the output. For example, an acceptable range of output is usually between 0 and 1, or it could be -1 and 1.

#### AdaBoost

AdaBoost, short for "Adaptive Boosting", which is a general ensemble method [34]. It focuses on classification problems and aims to convert a set of weak classifiers into a strong one. The final equation for classification can be represented by:

13 $$ F(x)=sign(\sum_{m=1}^{M} \theta_{m} f_{m}(x))  $$

where *f*_*m*_ represent the *m*^*th*^ weak classifier and *θ*_*m*_ is the corresponding weight. It is exactly the weighted combination of *M* weak classifiers.

#### Discriminant analysis

Discriminant analysis(DA) is one of the classification methods. The basic idea is that: two or more clusters or populations are priori known and one or more observations are classified into one of the known populations according to the measure characteristics [35]. Let *X* is a *q*-dimensional vector representing an observation from one of several possible classes. If the category is unknown, *X* can be classified using the discriminant analysis approach. Alternatively, it can be used to characterize the difference between classes via a discriminant function.

### Datasets

In order to evaluate the prediction performance of MMPCC on pseudo-negative sampling, we compare it with the state-of-the-art prediction methods. In experiments, we use four UCI Repository datasets [36] and three real bioinformatic datasets. Table 1 introduces the detail of the datasets.

**Table 1:** Description of datasets

Dataset	Positive	Negative	Attributes	Ratio
CMC	333	1140	9	3.4
Haberman	81	225	3	2.7
Solar Flare	69	1320	10	19.1
Oil	41	896	49	21.9
PDNA-543	9549	134995	180	14.1
PDNA-316	5609	67109	180	11.9
SNP	183	2891	25	15.7

From Table 1, we can see that the number of attributes of each dataset is 9, 3, 10, 49, 180, 180 and 25, respectively. We use all attributes of each dataset in MMPCC. In MMPCC, the Pearson correlation coefficient is used to calculate the similarity between negative and positive samples in Equation 7, and is also applied in Equation 10 and Equation 12. Additionally, the coefficient between two vectors *α*_*i*_ and *α*_*j*_ in Equation 5 is obtained by all attributes of each dataset.

In Table 1, Positive represents the number of positive samples, Negative represents the number of negative samples, and Ratio = Negative Numbers / Positive Numbers.

More specifically, the first UCI datasets, Contraceptive Method Choice (CMC) contains 333 minority samples and 1140 majority samples, and the number of attributes is 9. The second UCI datasets, Haberman’s Survival Data, contains 81 minority samples and 225 majority samples, and the number of attribute is 3. The third dataset Solar Flare records the number of solar flares. Each attribute calculates the number of a certain type of Solar Flare within 24 hours. Each instance represents the number of all types of flares in an active region on the sun. The data contains 69 minority classes and 1320 majority classes, with 10 attributes. The fourth datasets Oil contains 41 minority classes and 896 majority classes, including 49 attributes.

The first bioinformatic datasets, SNP data [37], included 183 positive samples and 2891 negative samples, and the number of attributes is 25. The second bioinformatic datasets, PDNA-543 [38], consists of 543 protein sequences, which are all related into the PDB (Protein Data Bank) before October 10, 2014. There are 9,549 DNA-binding residues as positive samples and 134,995 non-binding residues as negative samples in PDNA-543. The third bioinformatic datasets, PDNA-316, is constructed by Si *et al* 39], which has 316 DNA-binding protein chains and 5,609 binding residues and 67,109 non-binding residues.

### Evaluation metrics

In this study, four metrics are used to evaluate the performance of different classifiers, including Sensitivity (*Sen*), Specificity (*Spe*), Accuracy (*Acc*)and the Mathew’s Correlation Coefficient (*MCC*). They are calculated according to the following equations:

14 $$ Sen = \frac{TP}{TP+FN}  $$

15 $$ Spe = \frac{TN}{TN+FP}  $$

16 $$ Acc = \frac{TP+TN}{TP+FN+TN+FP}  $$

17 $$ \begin{aligned} &MCC = \\ &\frac{TP\bullet TN - FN\bullet FP}{\sqrt{(TP+FN)(TP+FP)(TN+FN)(TN+FP)}} \end{aligned}  $$

where *TP* is the number of true positives *TN* is the number of true negatives, *FP* is the number of false positives, *FN* is the number of false negatives, *P* is the number of positives, and *N* is the number of negatives.

Sensitivity indicates how well the test predicts the true positives, Specificity measures how well the test predicts the true negatives, *Accuracy* is expected to measure how well the test predicts both true positives and negatives, and *MCC* considers true and false positives and negatives. So, the higher the values of these evaluation metrics, the better the results.

## Results

The purpose of the evaluation is to examine the effectiveness of our proposed MMPCC method on selecting the pseudo-negative samples. Four sets of experiments are conducted. Experiment 1 compares the different percentage of pseudo-negative sampling on two UCI datasets. Experiment 2 compares the different percentage of pseudo-negative sampling on three bioinformatic datasets. Experiment 3 compares MMPCC with the max-relevance and the min-redundancy methods on the PDNA-316 dataset, which aims to evaluate the relation between the relevance and the redundancy. For simplicity, the max-relevance method is represented by MAXR and the min-redundancy method is represented by MINR. Experiment 4 compares MMPCC with other sampling methods on the bioinformatic datasets.

In experiments, five-fold cross-validation is used to train the dataset. In order to give comprehensive results, Discriminant Analysis, AdaBoost, Random Forest and Neural Networks are employed for classification. We use DA, Adaboost, RF and NN to represent these four classifiers in the experiments, respectively.

### Experiment 1: experiments on UCI datasets

This set of experiments examines the contribution of different percentage of pseudo-negative sampling on the UCI datasets [36]. The results are shown in Table 2 and Table 3. As mentioned previously, we use the metrics of *Sen*, *Spe*, *Acc* and *MCC*.

**Table 2:** Performance comparison of classifiers under different percentage of pseudo-negative samples on the CMC data

Percentage	Classifier	Sen(%)	Spe(%)	Acc(%)	MCC
0	DA	9.38	97.81	77.8	0.156
	AdaBoost	21.37	94.48	77.94	0.226
	RF	28.19	92.8	78.2	0.27
	NN	27.01	87.09	73.52	0.161
10	DA	17.6	94.85	75.7	0.198
	AdaBoost	25.76	93.58	76.78	0.266
	RF	39.22	91.77	78.75	0.369
	NN	40.92	86.98	75.55	0.302
20	DA	37.35	91.71	76.94	0.351
	AdaBoost	40.03	91.24	77.33	0.36
	RF	43.94	91.24	78.41	0.404
	NN	47.28	87.22	76.38	0.368
30	DA	52.46	88.34	77.8	0.438
	AdaBoost	50.89	88.83	77.67	0.431
	RF	50.87	89.98	78.48	0.448
	NN	53.39	87.86	77.73	0.439
40	DA	59.46	87.21	78.43	0.485
	AdaBoost	56.01	87.61	77.61	0.461
	RF	56.45	90.27	79.57	0.505
	NN	54.94	86.68	76.64	0.439
50	DA	66.78	85.42	79.08	0.530
	AdaBoost	64.01	87.37	79.42	0.531
	RF	62	88.71	79.63	0.532
	NN	61.02	87.38	78.42	0.505

**Table 3:** Performance comparison of classifiers under different percentage of pseudo-negative samples on the Haberman data

Percentage	Classifier	Sen(%)	Spe(%)	Acc(%)	MCC
0	DA	17.33	95.42	74.79	0.212
	AdaBoost	29.19	90.89	74.71	0.266
	RF	34.2	82.84	70.07	0.197
	NN	27.98	87.28	71.68	0.202
10	DA	21.77	93.96	72.96	0.236
	AdaBoost	32.72	86.12	70.58	0.214
	RF	33.38	83.91	69.38	0.197
	NN	30.37	82.01	67.04	0.144
20	DA	30.51	94.41	74.2	0.340
	AdaBoost	46.68	87.54	74.26	0.370
	RF	45.01	81.32	69.59	0.272
	NN	37.42	82.97	68.57	0.222
30	DA	31.73	95.1	73.32	0.36
	AdaBoost	51.81	87.15	75.65	0.422
	RF	51.06	79.6	70	0.311
	NN	42.39	84.54	70.36	0.291
40	DA	37.13	94.38	72.93	0.404
	AdaBoost	50.73	86.1	72.87	0.396
	RF	56.81	78.38	69.95	0.359
	NN	53.63	81	70.6	0.35
50	DA	38.61	93.83	71.74	0.405
	AdaBoost	61.46	82.26	73.81	0.447
	RF	60.75	78.22	70.95	0.395
	NN	52.41	79.81	68.56	0.339

Table 2 presents the performance of different classifiers on the CMC dataset, where the percentage of pseudo-negative samples changes from 0% to around 50%. 0% means the dataset is not used pseudo-negative sampling. We can see that the performance is improved with larger percentage of pseudo-negative samples, where the Random Forest method achieve 28.19%, 39.22%, 43.94%, 50.87%, 56.45% and 62% for *Sen* when the percentage of pseudo-negative samples is fixed to 0%, 10%, 20%, 30%, 40% and 50%, respectively. In addition, the *Acc* value is 78.2 *%*,78.75*%*, 78.41%, 78.48%, 79.57% and 79.63% and the *MCC* value is 0.27, 0.369, 0.404, 0.448, 0.505 and 0.532. The performance of different evaluation metrics show a trend of increasing with a higher percentage of pseudo-negative samples, which agrees with the real-world situation that: if we add more positive samples, the classifier will have better performance.

Furthermore, the Neural networks method achieves 27.01%, 40.92%, 47.28%, 53.39%, 54.94% and 61.02% for *Sen* when the percentage of pseudo-negative samples is fixed to 0%, 10%, 20%, 30%, 40% and 50%, respectively. Moreover, the *MCC* value is 0.161, 0.302, 0.368, 0.439, 0.439 and 0.505. For Discriminant analysis method, the *Sen* values are increased by 9.38%, 17.6%, 37.35%, 52.46%, 59.46% and 66.78% and the *MCC* values are increased by 0.156, 0.198, 0.351, 0.438, 0.485 and 0.530 on different percentage of pseudo-negative samples, respectively. Similarly, the performance of the AdaBoost classifier obtain improvement on *Sen* and *MCC*, which demonstrates the effectiveness of the proposed pseudo-negative sampling method.

Table 3 shows similar results on different metrics as Table 2, which verify that pseudo-negative sampling is very useful in classify the imbalance data and can obtain good performance of classification. Furthermore, the results indicates that pseudo-negative samples can be viewed as positive samples and be used to classify objects. For the instability of MMPCC, the results are often not unique in Table 3. There are three reasons about this issue: Firstly, four classification methods were employed, DA, RF, NN and AdaBoost in this study. Different machine learning method has different character, so the experiment results have little instability. Secondly, the value of Sensitivity and Specificity has little instability, but the value of *MCC* is more stable in most of experiments. As the Sensitivity and Specificity are the singular assessment metrics, *MCC* considers true and false positives and negatives and is generally regarded as a balanced measure. *MCC* can be used even if the class size is very different. Finally, the performance of different evaluation metrics shows a trend of increasing with a higher percentage of pseudo-negative samples.

### Experiment 2: experiments on real-Life bioinformatic datasets

**Fig. 2:**
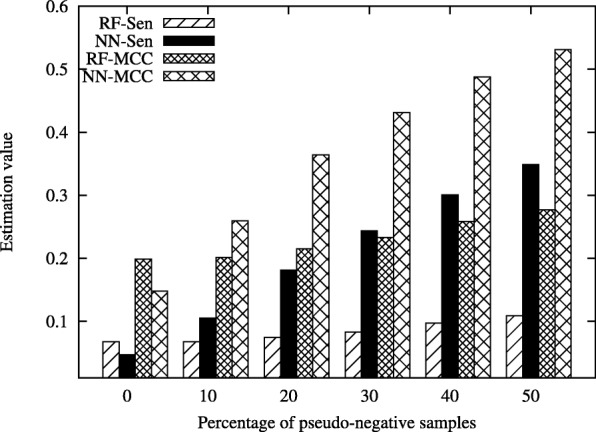
Performance comparison of RF and NN classifiers on PDNA-543 data under different percentage of pseudo-negative samples

**Fig. 3:**
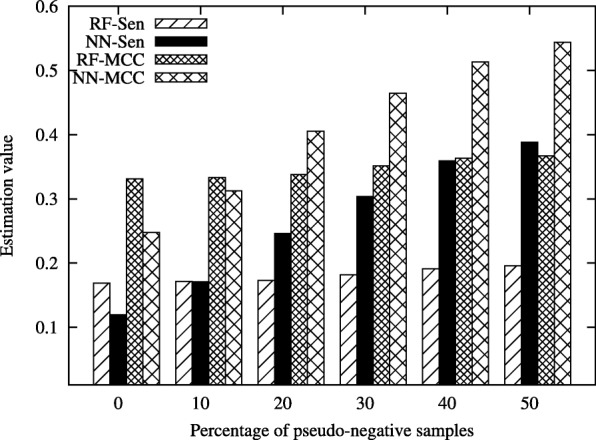
Performance comparison of RF and NN classifiers on PDNA-316 data under different percentage of pseudo-negative samples

In this section, we demonstrate the effectiveness of the proposed method, MMPCC, on the real bioinformatic datasets, including PDNA-543 [38], PDNA-316 [39] and SNP data [37]. The results are given in Fig. 1, Fig. 2 and Fig. 3.

Position Specific Scoring Matrix (PSSM) was used to extract the features from protein sequences of PDNA-543 and PDNA-316. PSSM is a very important type of evolutionary feature, which is obtained by running the PSI-BLAST program to search the SwissProt database via three iteration, with 10^−3^ as the E-value cutoff for multiple sequence alignment. In PSSM, there are 20 scores for each sequence position and each score implies the conservation degree of a specific residue type on that position. For each data instance, all the scaled scores in PSSM are used as its evolution features. In this study, we use the window size with 9 residues, and then obtain a vector of normalized PSSM scores whose dimensions of features are 9 ×20=180.

Figure 2 shows the classification performance on PDNA-543 dataset under different percentage of pseudo-negative samples, where RF-Sen and NN-Sen represent the Sensitivity value of RF and NN classifiers and RF-MCC and NN-MCC represent the MCC value of RF and NN classifiers.

The *Sen* and *MCC* metric of NN increase with the percentage of pseudo-negative samples changing from 0% to 50%. When the percentage of pseudo-negative samples changes from 0% to 30%, the *Sen* and *MCC* of RF algorithm keep unchanged. However, when the percentage of pseudo-negative samples is above 30%, there is a clear trend that RF has better performance as the percentage of pseudo-negative samples grows.

Figure 3 illustrates the classification performance on the PDNA-316 dataset under different percentage of pseudo-negative samples. The performance of RF is better than NN when the percentage is 0% and *%*10 in terms of *Sen* and *MCC*. When the percentage is above 20%, the performance of NN increases drastically and is better than RF, which shows that adding more pseudo-negative samples could help greatly improve the performance of classification. However, the performance of RF is almost unchanged. This is because the pseudo-negative samples has little effect on the RF algorithm in this dataset.

**Fig. 4:**
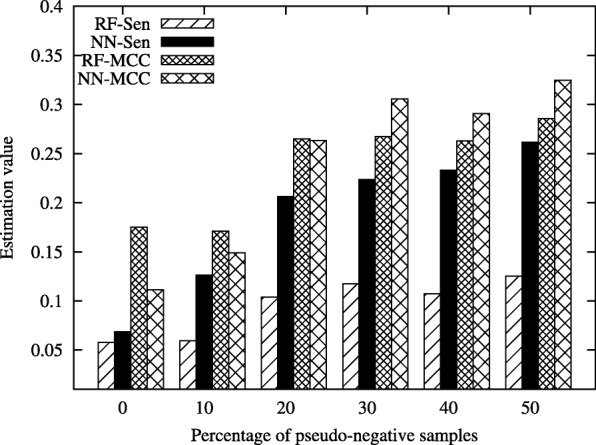
Performance comparison of RF and NN classifiers on SNP data under different percentage of pseudo-negative samples

Figure 4 shows the classification performance for data SNP on different percentage of pseudo-negative samples. The *Sen* of NN grows rapidly among different percentages of pseudo-negative samples and the *MCC* of NN gradually increases when the percentage changes from 0% to 30%, and then the fluctuate is small from 40% to 50%. We can find that the *Sen* and *MCC* of RF grows as the percentage of pseudo-negative samples gradually increases.

Generally speaking, this set of experiments illustrated that the pseudo-negative samples are very important and can be used to improve the effectiveness of classification.

### Experiment 3: comparison of mMPCC, mAXR and mINR on the pDNA-316 datasets

**Fig. 5:**
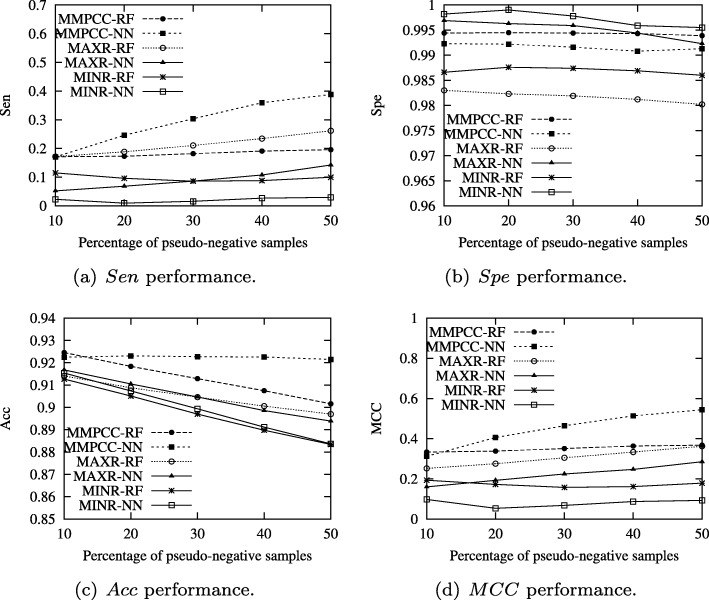
Comparison of algorithm MMPCC, MAXR and MINR on RF and NN classifiers for *Sen*, *Spe*, *Acc* and *MCC* performances

In this section, we employ the five-fold cross-validation to estimate the prediction performance of the proposed MMPCC method on four metrics. We compared MMPCC with other sampling methods including MAXR (max-relevance method based on Equation 7) and MINR (the min-redundancy method based on Equation 9) [30]. In experiments, the PDNA-316 dataset is employed to evaluate the effectiveness of MMPCC. The comparison results are shown in Fig. 5.

According to Fig. 5, it is straightforward to find that MMPCC outperforms the MAXR and MINR method in terms of *Sen*, *Spe*, *Acc* and *MCC* in the RF and NN classifiers. From Fig. 5(a), the pseudo-negative samples have a big influence on the *Sen* value. The *Sen* value of MMPCC is significantly better than MAXR and MINR, when NN is used as a classifier. For the RF classifier, MAXR is the best one when more pseudo-negative samples are added. By Fig. 5(b), with the increases of the percentage of pseudo-negative samples, the *Spe* value of MMPCC is very stable on RF and NN. This can be explained by the reason that some pseudo-negative samples are still negative ones. In addition, the *Sen* value can be improved with the cost of degradation of *Spe* value. Figure 5(c) demonstrates that the MMPCC method is the most stable method on *Acc* in the RF classifier. Figure 5(d) shows that the *MCC* value of MMPCC significantly outperforms the MAXR and the MINR methods. The performance of MAXR is better than MINR. The experimental results indicate that MMPCC attempts to utilize more representative samples and find the pseudo-negative samples (which can be viewed as positive samples) from the majority negative samples.

### Experiment 4: comparison of mMPCC and classical sampling methods on bioinformatic datasets

In order to verify the advantage of our method, we also compare the prediction performance of MMPCC with other classical over-sampling method, i.e., SMOTE method [40], on the PDNA-316 dataset.

SMOTE is an over-sampling approach in which the minority class is over-sampled by creating “synthetic” examples rather than by over-sampling with replacement. The minority class is over-sampled by taking each minority class sample and introducing synthetic examples along the line segments joining any of the *k* minority class nearest neighbors. Depending on the amount of over-sampling required, neighbors from the *k* nearest neighbors are randomly chosen. In order to compare the performance of the algorithm, we use the default value 5 nearest neighbors the same as the reference [40]. The results of comparison performance are shown in Table 4. Because neural network can learn and model the relationships between inputs and outputs that are nonlinear and complex, and make generalizations and inferences. The runtime performance of random forest is quite good, and they are commonly-used to deal with the unbalanced and missing data.

**Table 4:** Performance comparison between MMPCC and SMOTE under different percentage of pseudo-negative samples

Percentage(%)	Methods	Classifiers	Sen(%)	Spe(%)	Acc(%)	MCC
10	MMPCC	RF	17.13	99.44	92.46	0.333
		NN	17.05	99.23	92.25	0.312
	SMOTE	RF	16.01	98.27	91.34	0.235
		NN	5.2	99.69	91.68	0.16
20	MMPCC	RF	17.28	99.45	91.84	0.337
		NN	24.6	99.22	92.31	0.405
	SMOTE	RF	17.07	98.14	90.75	0.246
		NN	8.05	99.49	91.16	0.2
30	MMPCC	RF	18.18	99.44	91.29	0.351
		NN	30.38	99.16	92.27	0.464
	SMOTE	RF	17.69	97.95	90.08	0.25
		NN	10.16	99.23	90.5	0.216
40	MMPCC	RF	19.09	99.43	90.75	0.363
		NN	35.94	99.08	92.26	0.513
	SMOTE	RF	18.54	97.8	89.5	0.258
		NN	12.07	99.14	90.02	0.243
50	MMPCC	RF	19.56	99.39	90.16	0.367
		NN	38.82	99.13	92.15	0.543
	SMOTE	RF	18.5	97.72	88.9	0.258
		NN	14.05	99.01	89.55	0.266

According to Table 4, we can observe that MMPCC outperforms the SMOTE method in terms of all evaluation metrics. Taking *MCC* as an example, the MMPCC value in the NN classifier under different percentages of pseudo-negative samples are 0.312, 0.405, 0.464, 0.513 and 0.543, respectively, and the improvements are 0.152, 0.205, 0.248, 0.27 and 0.277, respectively compared to the SMOTE method. For other three evaluation metrics, the MMPCC method outperforms the SMOTE sampling method as well. As for RF classifier, Table 4 shows that the performance of MMPCC is better than that of the SMOTE method. As shown in Table 4, with the increase of percentage, the *MCC* value of the MMPCC in the RF classifier are 0.333, 0.337, 0.351, 0.363 and 0.367, respectively, and the improvements are 0.098, 0.091, 0.101, 0.105 and 0.109, respectively over the SMOTE method. This is due to the fact that a number of duplicated or artificial samples were introduced by over-sampling techniques for large-scale imbalanced data. But for MMPCC, there is no man-made duplicated data. In terms of the MMPCC sampling method, the pseudo-negative sampling technique helps identify more useful samples from the negative class which is often neglected, so it performs better than the SMOTE sampling method.

### Experiment 5: experiments on highly imbalance ratio datasets

In order to validate the performance of the proposed method on highly imbalance Ratio datasets, the comparative evaluation on two UCI datasets, Solar Flare and Oil, are performed. The dataset Solar Flare contains 69 minority classes and 1320 majority classes; with 10 attributes, and the Ratio is 19.1. The Oil dataset contains 41 minority classes and 896 majority classes, including 49 attributes, and the Ratio is 21.9.

Table 5 demonstrates the classification results of the Solar Flare dataset with highly imbalance Ratio. Overall, the performance is increased with a larger percentage of pseudo-negative samples.For example, the random forest method obtain 1.43%, 4.00%, 13.53%, 25.03%, 20.68% and 32.57% for *Sen* as the percentage of pseudo-negative samples is fixed to 0%, 10%, 20%, 30%, 40% and 50%, respectively. Moreover, the *MCC* value is 0.01, 0.06, 0.23, 0.39, 0.32 and 0.44. For the neural networks method, the *Sen* values are increased from 7.25%, 8.01%, 20.88%, 32.03%, 28.33% to 35.48% and the *MCC* values are increased from 0.05, 0.06, 0.24, 0.33, 0.28 to 0.38 on different percentage of pseudo-negative samples. We can conclude that the performances of different evaluation metrics show a significant improvement with a higher percentage of pseudo-negative samples, even in the situation of highly imbalance Ratio.

**Table 5:** Classification results of the Solar Flare dataset with highly imbalance Ratio

Percentage	Classifier	Sen(%)	Spe(%)	Acc(%)	MCC
0	RF	1.43	99.02	94.24	0.01
	NN	7.25	96.90	92.51	0.05
10	RF	4.00	99.16	94.02	0.06
	NN	8.01	96.73	91.94	0.06
20	RF	13.53	99.39	94.31	0.23
	NN	20.88	97.63	93.09	0.24
30	RF	25.03	99.08	94.39	0.39
	NN	32.03	97.08	92.95	0.33
40	RF	20.68	98.92	93.59	0.32
	NN	28.33	96.44	91.79	0.28
50	RF	32.57	98.84	93.95	0.44
	NN	35.48	97.05	92.51	0.38

Table 6 demonstrates the classification results of the Oil dataset with highly imbalance Ratio. From the Table 6, the random forest method achieves 14.50%, 19.60%, 33.53%, 39.83%, 50.36% and 49.76% for *Sen* when the percentage of pseudo-negative samples is fixed to 0%, 10%, 20%, 30%, 40% and 50%, respectively. In addition, the *MCC* value is 0.27, 0.32, 0.43, 0.50, 0.63 and 0.59. For the neural networks method, the *Sen* values are increased from 52.18%, 51.95%, 41.26%, 45.83%, 54.96% to 48.09% and the *MCC* values are increased from 0.58, 0.54, 0.48, 0.51, 0.55 to 0.51 with different percentage of pseudo-negative samples. It indicates that the proposed method is prone to improve the discrimination of minority class while retains the considerable stability.

**Table 6:** Classification results of the Oil dataset with highly imbalance Ratio

Percentage	Classifier	Sen(%)	Spe(%)	Acc(%)	MCC
0	RF	14.50	99.68	96.07	0.27
	NN	52.18	98.90	96.83	0.58
10	RF	19.60	99.55	95.74	0.32
	NN	51.95	98.54	96.37	0.54
20	RF	33.53	98.98	95.60	0.43
	NN	41.26	98.65	95.72	0.48
30	RF	39.83	98.99	95.65	0.50
	NN	45.83	98.29	95.32	0.51
40	RF	50.36	99.31	96.26	0.63
	NN	54.96	97.83	95.08	0.55
50	RF	49.76	98.75	95.52	0.59
	NN	48.09	97.84	94.58	0.51

**Fig. 6:**
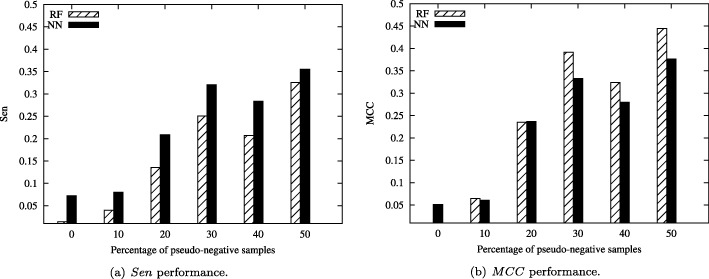
Classification results of the Solar Flare dataset with highly imbalance Ratio for *Sen* and *MCC* performances

Furthermore, Fig. 6 shows the classification performance on the Solar Flare dataset under different percentage of pseudo-negative samples. From Fig. 6(a), the Sen metric of neural network increase with the percentage of pseudo-negative samples changing from 0% to 50%. Even there is little fluctuation from 40% to 50%. It maybe the distribution of original dataset is unclear. In the future, we will consider how to choose the percentage of pseudo-negative samples automatically. For MCC performance, similar phenomenon can be obtained from Fig. 6(b).

**Fig. 7:**
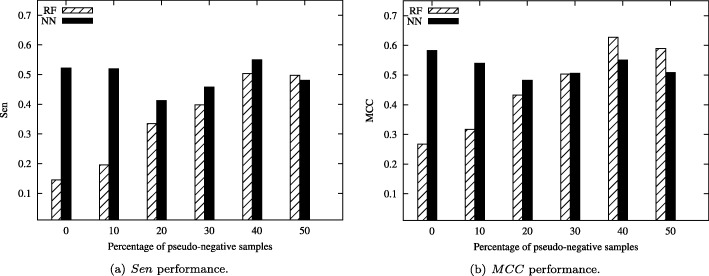
Classification results of the Oil dataset with highly imbalance Ratio for *Sen* and *MCC* performances

Figure 7 shows the tendency of Oil dataset with highly imbalance Ratio in neural network and random forest classification. We can see that Sen and MCC of random forest gradually increase when the percentage changes from 0% to 50% in Fig. 7(a). However, the value of Sen and MCC of neural network has some fluctuate from 0% to 50%. It indicated that random forest is more stability of the proposed method for this dataset. Similar trends of MCC performance can be obtained from Fig. 7(b).

## Discussion

Here we designed a supervised learning method based on max-relevance and min-redundant criterion beyond Pearson correlation coefficient and tested on four UCI datasets and three real bioinformatics datasets. Our results indicated that MMPCC is better than other sampling methods in terms of several evaluation metrics. The performance of different evaluation metrics shows a trend of increasing with a higher percentage of pseudo-negative samples. On the other hand, different machine learning method has different character, so the experiment results have little instability. We also observed that MMPCC method can have good performance even in the situation of highly imbalance Ratio. This reveals that pseudo-negative samples are good at solving the imbalance dataset problem.

## Conclusions

In this study, we propose a new sampling method, which is called pseudo-negative sampling, to handle the imbalanced classification problem based on Pearson correlation coefficient which integrates the max-relevant and min-redundant. In addition, an incremental searching method is used to find the target sample with little cost of computation. The experimental results demonstrate the superior performance of our method compared to other algorithms for imbalanced classification problems.

In future, we will apply the proposed MMPCC algorithm in more real-world bioinformatic applications with large-scale imbalanced data. We will investigate the possibility of extending the MMPCC method to handle multiple-classification problem. Furthermore, we will use the state-of-the-art machine learning methods [41*–*46] to handle the imbalanced classification problem.

## Data Availability

All relevant data are included in this published article and its additional files. The datasets used and/or analyzed during the current study are available from the corresponding author on reasonable request.

## Abbreviations

Acc: Accuracy
CMC: Contraceptive method choice
FN: The number of false negative
FP: The number of false positive
MAXR: Max-relevance method
MCC: Mathews correlation coefficient
MINR: Min-redundancy method
MMPCC: Max-relevance and min-redundancy Pearson correlation coefficient
PCC: Pearson correlation coefficient
Pre: Precision
PSSM: Position specific scoring matrix
Sen: Sensitivity
Spe: Specificity
TN: The number of true negatives
TP: The number of true positive
